# Bacterial Chemotaxis in an Optical Trap

**DOI:** 10.1371/journal.pone.0018231

**Published:** 2011-04-08

**Authors:** Tuba Altindal, Suddhashil Chattopadhyay, Xiao-Lun Wu

**Affiliations:** Department of Physics and Astronomy, University of Pittsburgh, Pittsburgh, Pennsylvania, United States of America; New Mexico State University, United States of America

## Abstract

An optical trapping technique is implemented to investigate the chemotactic behavior of a marine bacterial strain *Vibrio alginolyticus*. The technique takes the advantage that the bacterium has only a single polar flagellum, which can rotate either in the counter-clock-wise or clock-wise direction. The two rotation states of the motor can be readily and instantaneously resolved in the optical trap, allowing the flagellar motor switching rate 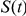 to be measured under different chemical stimulations. In this paper the focus will be on the bacterial response to an impulsive change of chemoattractant serine. Despite different propulsion apparati and motility patterns, cells of *V. alginolyticus* apparently use a similar response as *Escherichia coli* to regulate their chemotactic behavior. Specifically, we found that the switching rate 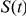 of the bacterial motor exhibits a biphasic behavior, showing a fast initial response followed by a slow relaxation to the steady-state switching rate 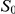. The measured 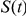 can be mimicked by a model that has been recently proposed for chemotaxis in *E. coli*. The similarity in the response to the brief chemical stimulation in these two different bacteria is striking, suggesting that the biphasic response may be evolutionarily conserved. This study also demonstrated that optical tweezers can be a useful tool for chemotaxis studies and should be applicable to other polarly flagellated bacteria.

## Introduction

Microorganisms face many challenges in their natural habitats, and they develop different strategies to adapt to the environment they live in. One of the challenges for these microorganisms is to identify what is good or bad for them and respond appropriately. Thus far the best studied case is the chemotactic behavior of enteric bacterium *Escherichia coli*. This bacterium uses the run-tumble swimming pattern to navigate in an environment, i.e., when the temporal signal is favorable the run interval is lengthened, but when the signal is unfavorable the run interval is shortened. Thus by regulating the length of the swimming intervals, the bacterium executes a biased random walk, directing it towards the source of attractant or away from a repellent. However, not all bacteria live in conditions similar to *E. coli*, and it is of great scientific interest to learn and understand how other diverse bacterial species handle challenges in a variety of environments. In this study we report new findings of bacterial chemotaxis of *Vibrio alginolyticus*. This bacterium lives in the ocean, but it has much in common with *E. coli* such as its physical size and its motility being powered by rotary motors. Unlike *E. coli*, however, the flagellar motor of *V. alginolyticus* is more powerful, which can rotate at an angular frequency of a few kilohertz, pushing the cell body at a speed 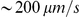 [1]. These values are nearly ten times of those typically seen *in E. coli* [2], [3], reflecting the different ecosystems the two bacteria inhabit. Another significant difference between the two bacteria is that *V. alginolyticus* possesses only a single polar flagellum when it is grown in a liquid medium. This suggests that forward and backward swimming paths are time-reversal symmetric when the motor reverses its direction [4]. Our recent study, however, demonstrates that *V. alginolyticus* incorporate an additional movement, which we call a flick, that randomizes cells' swimming trajectories. The flick occurs specifically at the transition from clockwise (CW) to counter-clockwise (CCW) rotation, or from backward to forward swimming, and is almost instantaneous [5]. In the light of these physiological differences (polar vs. peritrichous flagellation) and their varied motility patterns, one wonders if there is also a difference in the way the flagellar motor is regulated by the internal chemotaxis network.

**Figure 1:**
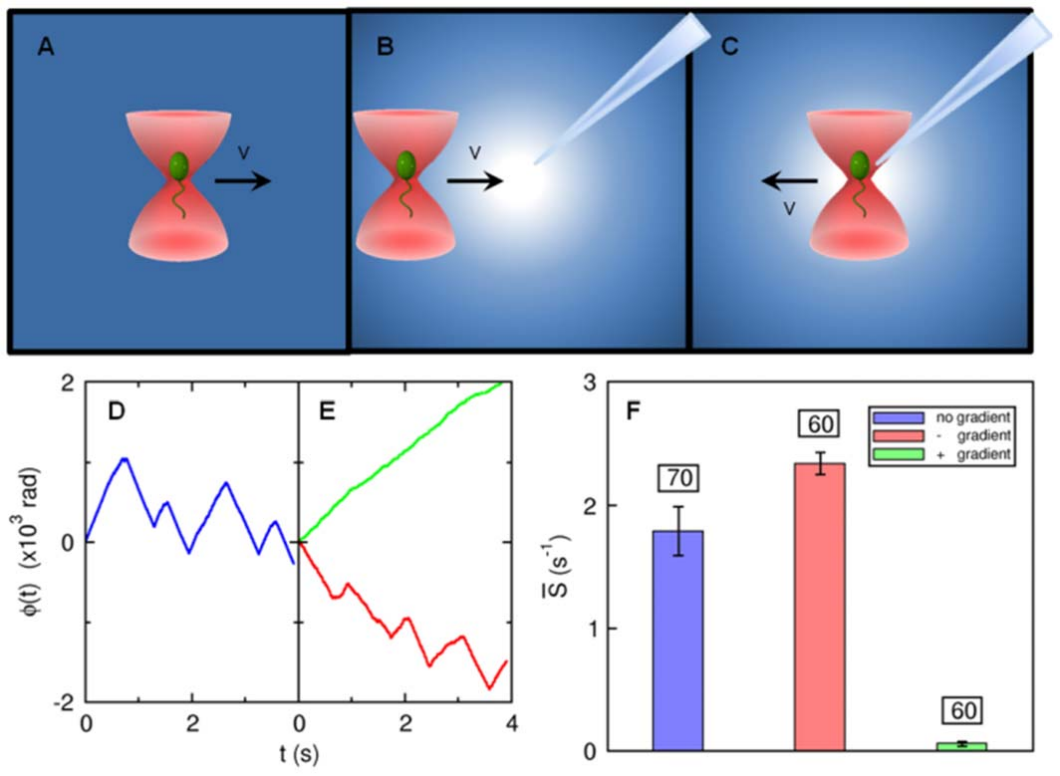
Probing bacterial chemotactic response with an optical tweezers. To investigate cell's response to a chemoattractant gradient, a micropipette filled with 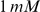 of serine was used. The concentration profile is determined by molecular diffusion [41]. (A) is a control experiment in which a *V. alginolyticus* cell was dragged at a speed 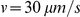 in a uniform TMN buffer to obtain its steady-state switching rate. In (B), the cell was trapped 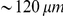 away from the tip and then dragged towards it for 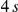 at the same speed. In (C), a cell was initially trapped at a distance 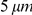 from the tip and was then dragged away from it for 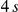 at the same speed. In (D), the flagellar motor rotation angle (or the winding angle) as a function of time 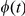 is measured in the optical trap when the trapped cell was moved in the motility buffer without chemoattractant. In (E), the bacterium was moved towards (green) and away from (red curve) the source of attractant. In the homogeneous medium (D), the motor reverses its direction roughly once every 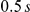. However, when the cell is moving up the gradient (green in (E)) the motor reversal is completely suppressed. When the same cell was moved down the gradient, frequent motor reversals from CW
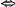
CCW were again observed. In (F), the average switching rates 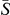 for the three different stimuli are displayed. The blue bar is for the steady-state case, while the green and the red bars are for cells moving up and down the gradient, respectively. We noticed that there was only a small difference when the cell was forced to move away from the source compared to the steady-state case. The error bars are standard errors of the mean calculated based on the cell numbers indicated above the bars.

A major difficulty in studying chemotactic behavior of *V. alginolyticus* is that the classical rotation assay [6], [7] that has been used successfully for *E. coli* cannot be reliably applied to this marine bacterium. This perhaps is due to the membrane sheath that covers the flagellum, making it difficult to tether to a substrate [8]. We overcame this difficulty by developing an optical trapping technique to monitor the rotation of the flagellar motor [3]. The optical trap can hold the bacterium in place without restricting its rotational motion. As illustrated in Figs. 1(A to C), the trapped bacterium can be forced to move in a homogeneous medium (A), towards a chemical source (B), or away from it (C), while the state of motor rotation is monitored continuously at a high rate by a photo-diode. The measurements can achieve a high signal-to-noise ratio owing to the fact that *V. alginolyticus* has a single polar flagellum such that rotation of the cell body reacts instantaneously to the flagellum rotation. By way of introduction, Figs. 1(D, E) display the response of a bacterium when subjected to the manipulations as described in Figs. 1(A to C). The chemical source in this case is created by a micropipette filled with 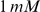 of serine, which is an attractant to *V. alginolyticus*, and the flagellar rotation (or the winding) angle 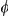 is recorded as a function of time 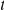. As shown, when the bacterium is in a homogeneous medium, which can be called a steady state, the winding angle 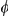 fluctuates in time, giving rise to a saw-tooth functional form as displayed by the blue curve in (D). When the cell is moved towards the source, 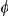 increases steadily, indicating no motor switching as displayed by the green line in (E). In contrast, when the cell is moved away from the source, 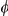 fluctuates wildly as illustrated by the red curve in (E). We noticed that the positive and negative slopes of 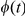 are about the same in magnitude, indicating that the flagellum rotation angular velocities 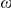 are similar for CCW and CW directions. Moreover, the motor reversal is almost instantaneous, i.e., within the resolution of our measurement, no obvious delays or pauses can be detected during a reversal. The above measurements can be repeated for a large number of bacteria, which allow the time-dependent switching rate 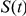 to be determined after an ensemble average.

A nice feature of our technique is that it permits experimenters to design paths for a cell so that the chemical signal 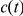 it receives can be predetermined. This potentially enables detailed studies of bacterial chemotactic response to a variety of stimulation patterns that have only been achieved in tethered *E. coli* cells with the help of a programmable mixing apparatus [9]. Our optical trapping technique is general, since it does not rely on cell tethering, and therefore should be applicable to different bacterial species.

In this work, we investigated the simplest stimulation, where 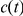 is approximately 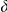 in time and its amplitude was varied systematically. We found that the response of the bacterium is biphasic in a manner similar to *E. coli*. However, the excitation time 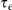 and the adaption time 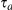 are both very short with 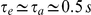. Biologically, these time scales may be associated with the dephosphorylation time 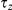 of response regulators CheY-P and the methylation time 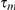 of chemoreceptors (or MCPs), similar to *E. coli*. Thus, an important finding of this experiment is that upon a brief stimulation, the chemotaxis network of *V. alginolyticus* appears to employ only a single time scale for chemosensing.

## Results

### A. Bacterial motion in the optical trap

**Figure 2:**
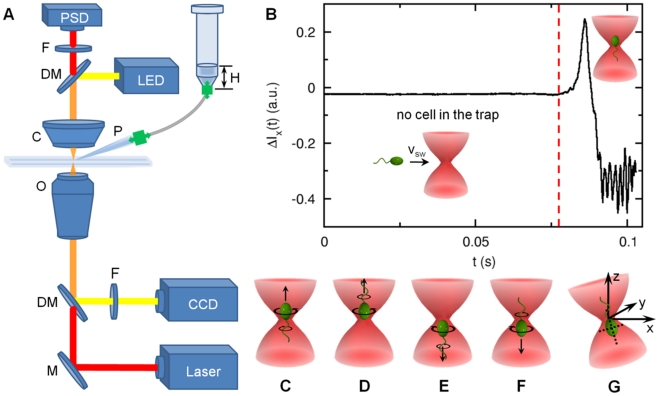
Experimental setup. (A) The trapping beam (red) from an IR laser was focused into the sample chamber by a high N.A. objective (O). The scattered light was refocused on to a position-sensitive detector (PSD) using a high N.A. condenser (C). An infrared filter (F) was placed before the PSD to cut off ambient light. The focal plane was illuminated by a LED and imaged by a CCD camera via dichroic mirrors (DMs). To eliminate the laser light, a visible band-pass filter was used in front of the CCD. To stimulate a trapped cell, a micropipette (P) was mounted onto the stage that held the sample chamber. The x–y stage movements were controlled by DC actuators whereas the z movement was controlled by a piezo-actuator. A small hydrostatic pressure was applied to the micropipette via a plastic tubing by a water column of height 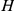, where the plastic tubing was filled with air. (B) When the bacterium was outside the optical trap, the optical signal 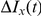 was quiescent. However, when the bacterium swims into the optical trap, it first produces a large spike in 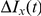 and then the signal fluctuates with a large amplitude. The red line in the figure indicate the moment just before the bacterium falls into the optical trap. We used the change in the rms value of 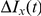 to trigger the movement of the x–y stage, causing a relative motion between the trapped cell and the micropipette tip. A trapped bacterium can assume one of the four configurations (C to F) and its swimming direction cannot be resolved. (G) In a slightly tilted optical trap, the 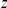 position is coupled to the 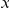 position and thus the CCW and CW rotation of the motor can be readily measured by the PSD. As discussed in Materials and Methods, this tilted optical trap significantly improves the detection of a motor reversal, but it still does not resolve degeneracies in the cell orientation as displayed in (C to F).

**Figure 3:**
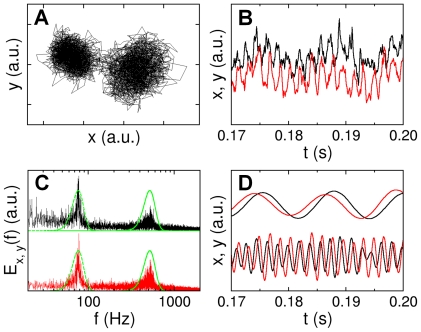
Bacterial positions in the optical trap. The bacterial position 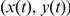 in the optical trap is recorded by the PSD, and a trace of 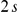 is given in (A). A stretch of the data for the x- (black) and y-channel (red) is given in (B), and the corresponding power spectra are presented in (C). The peaks in the power spectra are due to cell-body and flagellar rotations. We applied Gaussian band-pass filters (green lines) to 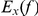 and 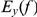 to separate rotational motions of the cell body and the flagellum. The filtered data can be used to perform an inverse Fourier transformation, yielding the results for the cell-body (top) and the flagellum rotations (bottom) in (D). Note that after band-pass filtering the phase differences between red and black curves for the cell body (top) and the flagellum (bottom) are opposite to each other, indicating that the cell body and the flagellum are rotating in opposite directions. When a polar angle is used, the angular displacements (or the winding angle) 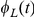 of the cell body and the flagellum 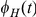 can be calculated. In this paper, we exclusively use 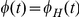.

**Figure 4:**
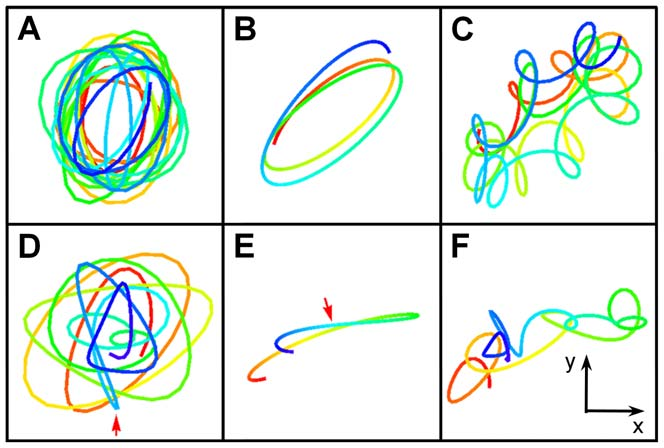
Lissajous figures of bacterial trajectories in the optical trap. For illustration purpose, the top and bottom rows depict two bacterial trajectories (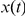, 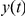) in the optical trap, lasting for 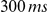 and 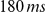 each. Here, (A) and (D) correspond to the high frequency 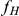 components of rotation; (B) and (E) correspond to the low frequency 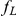 components of rotation; and (C) and (F) are the linear superposition of (A) and (B), and (D) and (E), respectively. In all of these figures, the flow of time is designated by colors, starting with red and ending with blue. We note that the sense of rotation is opposite for the high and the low frequency components, which is expected for the torque balance between the bacterial cell body and flagellum. For the Lissajous figures in the lower row, a switching event occurs at the location (see arrows) where the color turns from green to blue.

Our measurements were carried out in a home-built optical tweezers (see Fig. 2A), which has been described in details in Ref. [3]. A brief description of the setup is also provided in Materials and Methods. Using radiation pressure from a tightly focused infrared laser, a bacterium can be held in place or be moved about without restricting its rotational degrees of freedom. The cylindrical shape of the bacterium ensures that once trapped, its cell body is aligned with the optical axis of the trap as illustrated in Figs. 2(C to F). Waves due to flagellar rotation propagate along the cell body, causing its center of mass position (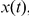 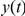) to fluctuate, which can be interrogated using a two-dimensional position sensitive detector (PSD). Fig. 3A displays a typical time trace (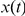, 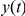) for a trapped bacterium. The bacterial trajectory in the optical trap is concentrated in two lobes, which correspond to the rotational states of the motor (see more discussions below). A short segment in one of the lobes is plotted against time as displayed in (B) for 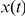 and 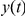, corresponding to the black and red curves, respectively. The power spectra 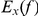 and 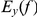, corresponding to fluctuations in 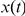 and 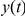, are given in (C). Here, one observes two sharp peaks located at 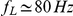 and 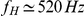. These frequencies are due to the rotation of the cell body 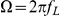 and the flagellum 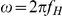, respectively. One can apply band-pass filters (see dotted green curves) to extract the slow and the fast rotations of the cell body as depicted in Fig. 3C. We applied Gaussian filters that are centered at the peaks and with a width of 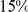 of the peak frequency. One observes in Fig. 3D that after filtering the slow cell-body rotation and the fast flagellum rotation are rather regular. Moreover, there is a phase difference between 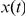 and 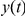 traces, and this phase difference is opposite for the fast and slow rotations, indicating that the cell body and the flagellum rotate in the opposite directions. The filtered data for the 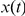 and 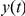 displacements can be recombined to produce Lissajous figures, which are displayed in Fig. 4. Here, the left column (A and D) is for the high-frequency (flagellum) rotation, the middle column (B and E) is for the low-frequency (cell body) rotation, and the right column (C and F) is for the linearly superimposed rotations of both fast and slow components. The time is color coded with red being the beginning and blue the end of the trajectory. A convenient way to characterize the state of the flagellar motor is to use the winding angle 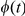, which as delineated in Fig. 1 allows the motor reversals to be characterized.

**Figure 5:**
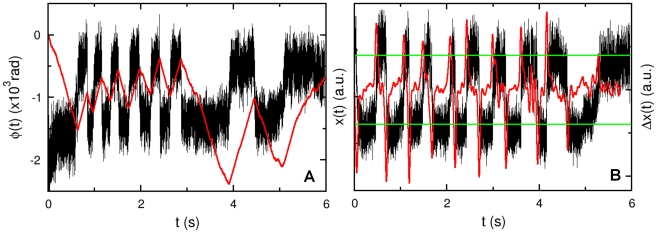
Two rotation states of bacterial flagellar motor in the optical trap. In (A), the correlation between 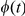 and 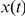 is demonstrated. The x-channel, 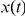, from the PSD exhibits a two-state behavior as displayed by the black curve. For a given state, the thick dark band corresponds to rapid oscillations due to cell-body and flagellum rotations as delineated in Fig. 3. The transition from one state to the other is due to motor reversals. These transitions are strongly correlated with the turning points in the angular displacement 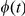 depicted by the red curve. In (B), the switching events occurring at different times are identified in a typical run. The smoothed time derivative 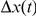 (red) is obtained by convolving 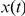 (black) with the derivative of a Gaussian function. The width of the Gaussian is adjusted such that it captures the changes occurring over times greater than 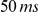. Only those events for which the derivative exceeds the threshold (green lines) are registered as switching events. The threshold is determined individually for each cell.

It must be pointed out that while the 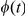 measurement is straightforward, it works the best for cells that display wobbly swimming patterns, i.e., the cell body spirals about the swimming direction. For cells that are not wobbly, such as those with high axial symmetry, the signal in the PSD is small and it sometimes becomes difficult to determine a motor reversal unambiguously. A simple solution to this problem is to tilt the laser trap slightly so that the 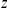 movement is coupled to the 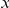 movement, which can be detected by the PSD. Fig. 2G depicts the optical tweezers setup where a bacterium is assumed to be trapped in the tail-up position. The CCW (CW) rotation of the flagellum will push (pull) the cell body so that it gives a small displacement in the positive (negative)×direction (see more details in Materials and Methods). Fig. 5A displays the switching events using this technique. As can be seen the correlation between the 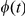 and the 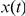 measurements is nearly perfect. Our current experimental setup would not allow us to distinguish the rotation directions of a flagellar motor (see more discussions in Materials and Methods); therefore, only measurements concerning the motor switching rate 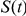 will be reported. For a bacterium performing 3-step motility pattern with the mean forward and backward swimming times being about the same [5], 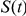 is a relevant quantity for characterizing its chemotactic behavior.

### B. Characterization of the chemoattractant concentration profile

**Figure 6:**
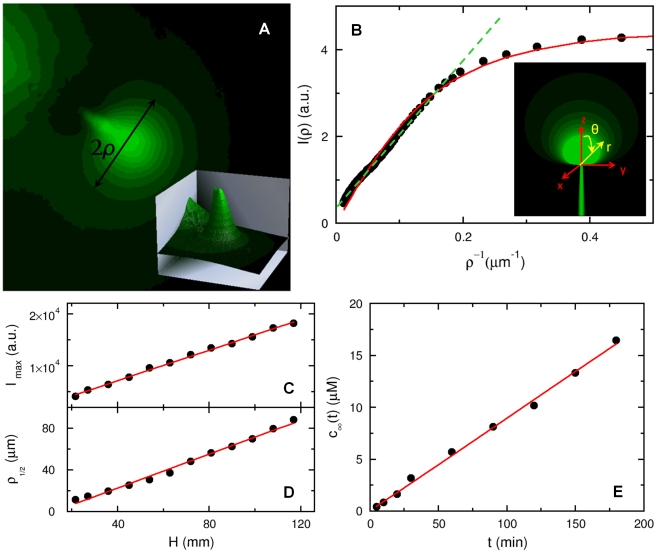
The chemoattractant concentration profile. (A) To visualize the concentration profile of serine, a micropipette was filled with 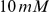 fluorescein and a small hydrostatic pressure was applied by a water column to maintain a continuous flow of dye into the sample chamber. The height of the water column was set to 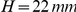 and kept fixed in all measurements. The fluorescence intensity distribution after the background subtraction is displayed in (A) and in the inset. In (B), the measured intensity 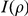 is plotted against 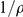, where 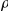 is along the radial direction as delineated in (A). In the far field, 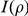 is proportional to 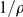 as displayed by the dashed green line, which is expected from the calculation. The solid line is the fit to Eq. 6, which captures both the near- and far-field behaviors. The inset is a computer generated plot of the dye distribution according to Eq. 3, where 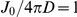 and the same coordinate system is used as in the calculation. In (C and D), the fluorescence peak intensity (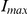) and the half-width at half-height (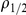) were measured as a function of the water column height 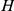. In (E), the micropipette was filled with 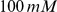 of fluorescein, and the mean fluorescein concentration in the sample chamber (with a total volume of 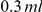) was measured as a function of time 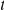.

A stable serine concentration gradient was established following the procedure described in Materials and Methods. Fig. 6A displays a background corrected fluorescence intensity profile of fluorescein, which mimics serine. We can model the concentration profile 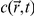 using the diffusion equation,
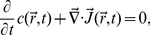
(1)where 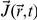 is the flux. Phenomenologically, we write the flux as 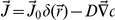, which consists of a deterministic injection term and a term due to thermal diffusion. For simplicity of calculation, the injection term is approximated by a 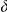 function because the mouth of the capillary is very small. In the above, 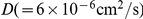 is the diffusion constant of the dye (or 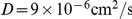 for serine) and 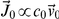 with 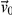 being the injection velocity. The proportionality constant between 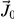 and 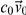 has a dimension of length to the cubic power. We seek the steady-state solution, which is given by
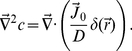
(2)Using the mathematical identity 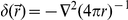, Eq. 2 can be solved with the result,
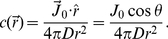
(3)


We note that this concentration profile is different from when 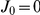. In that case, the quasi-steady-state profile is determined by thermal diffusion alone, and the profile at large distances decays as 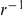 [10]. A computer generated dye distribution according to Eq. 3 is given as an inset in Fig. 6B. The video images acquired using the CCD camera are two dimensional, and thus the above calculated three-dimensional concentration profile needs to be integrated over the depth of the visual field in order to compare with the measurement. For simplicity, we assumed that 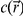 is viewed along the 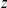-axis (see Fig. 6B), and our measured intensity profile 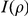 with 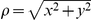 is proportional to the two-dimensional projection of 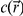 onto the 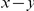 plane according to,
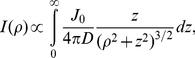
(4)where 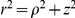 and 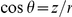 defined in the inset of Fig. 6B. The above integration yields,
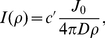
(5)where 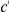 is a constant that can be determined by calibration. However, in this work this is not important since we are only interested in the width of the concentration profile. As depicted in Fig. 6B, where 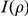 vs. 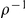 is plotted, our theoretical result (dashed line) agrees well with the measurement (solid circles) over a broad range of 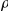; the graph displays a quasi linear region for small 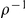 that is expected from Eq. 5. The strong deviation from the linear behavior occurs when 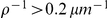, which is also expected because near the mouth of the micropipette the flux 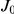 cannot be simply described by the 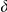 function. To remove the singularity at 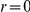 in Eq. 3, one can replace 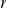 by 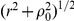, which leads to
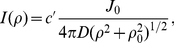
(6)after the 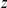 integration. This equation is used to fit the measured intensity profile in Fig. 6B as displayed by the solid red line. The fitting procedure yields 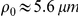, which can be considered as the width of the concentration profile in our experiment. Measurements using different hydrostatic pressures 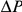 show that the intensity maximum 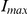 at the center of the concentration profile is a linear function of 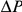, which is displayed in Fig. 6C along with the fitting line. This linear dependence is expected from Eq. 5 since the injection velocity 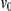 or the rate 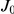 is proportional to 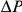 according to the Stokes law [11]. In the experiment, 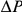 is controlled by a water column of height 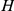, as delineated in Fig. 2. In Fig. 6D, we also plotted the half-width 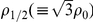 at half-height 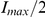 as a function of 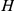. Here again 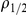 is approximately linear in 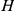. For the measurement presented below we set the water column height at 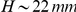, which yields 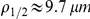 (or 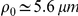).

Although the concentration profile 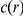 is established by injection, the attractant flux is so small that the background serine concentration increases negligibly during the measurement, which lasts less than an hour. A control experiment was conducted in the same sample chamber with 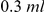 of TMN motility buffer (see Materials and Methods), and the micropipette was filled with 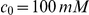 of fluorescein. A small volume of fluid inside the chamber was sampled periodically after thorough mixing, and its fluorescence intensity was determined by a fluorescent spectrometer (Perkin Elmer, LS-3B). This measurement, which is presented in Fig. 6E, yields 
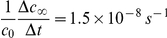
, where 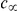 is the background fluorescein concentration in the chamber after mixing. In our stimulation experiment, the highest serine concentration used was 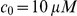, which corresponds to a total flux of 
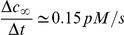
. For 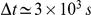, 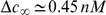. This change is significantly less than the stimulation level 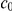, or the sensitivity of *V. alginolyticus* to serine, which we show below to be 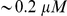.

### C. The average switching rate

As a demonstration of our technique, Fig. 1B displays a simple measurement where individual bacteria were trapped at a distance 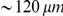 from the tip of the capillary filled with 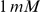 of serine. The cells were then moved towards the tip or up the gradient direction (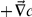) at a speed of 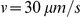. The average switching rate among 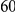 cells were determined. Likewise, a similar number of bacteria were also trapped at 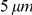 from the capillary tip (see Fig. 1C) and moved away from the tip (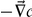) at the same speed. These two sets of measurements were presented in Fig. 1F by the green and the red bars, respectively. As a comparison, we also trapped a group of 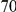 bacteria and moved them in a homogeneous TMN background (see Fig. 1A). This measurement is displayed by the blue bar in the same figure. The data showed that upon moving away from the source of attractant, the average switching rate 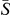 increases compared to that in the homogeneous TMN. A striking feature of Fig. 1F is that when the cells were moved towards the source of attractant, 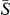 is suppressed to such an extent that it is barely measurable. For instance, among the 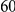 cells tested, only 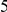 showed a motor reversal when moved up the gradient. These results are consistent with that displayed in Fig. 1E (see the green line). Our measurements indicate that (i) the response of *V. alginolyticus* to a deteriorated and an improved environment is not symmetrical; it appears that cells can more readily suppress the motor switching rate than enhancing it. (ii) Since the cell orientation in the optical trap is random when the motion of the trap is initiated, it can be concluded that this suppression must take place in either cell orientation. Hence, the cells of *V. alginolyticus* must perform chemical sensing all the time with a 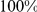 duty cycle. It also implies that the switching logic of *V. alginolyticus* is different from *E. coli* in that the former lengthens both of its CCW and CW intervals but the latter only lengthens its CCW interval when stimulated by an attractant.

### D. The time-dependent switching rate

#### D1. Chemotactic response measurements

A more revealing quantity to measure is the time dependent switching rate 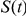 when the cells are exposed to a short pulse of stimulus at 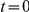. In order to measure this quantity reliably, it is crucial to have precise timing. As discussed in Materials and Methods, there is a considerable change in the optical signal when a bacterium becomes trapped. This signal provides a convenient means for us to define 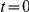 and to synchronize all the subsequent steps, which include the movement of an x–y stage, monitoring the position of the cell in the optical trap using PSD, and termination of the run. Specifically, once a bacterium falls into the optical trap that is located 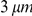 from a serine-filled micropipette tip, it is forced to move away from the tip with a speed 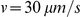 that is comparable to the swimming speed 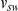 of the bacterium. Since the width of the serine profile is 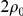, the characteristic time of a cell's exposure to the chemical is 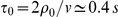. To obtain 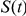, we first identified, for each bacterium trapped, the times when individual switches took place. This was accomplished by accentuating the switching events using a smoothed time derivative of 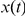 and a threshold was then applied as shown in Fig. 5B. The smoothed time derivative was performed by convolving 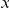
(t) with the derivative of a Gaussian function,
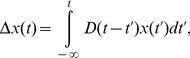
(7)where 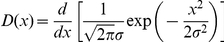. The distance between the positive and negative peaks of the kernel was set to 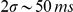 so that the convolution is equivalent to a finite-time difference (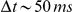) with low-pass filtering to get rid of high frequency oscillations of the cell body. Fig. 5B displays the original time series 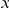
(t) (black) of a typical cell along with its smoothed time derivative 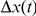 (red). We chose a threshold manually for each cell so that all the major abrupt changes in the derivative were accounted for. This is illustrated by the two green lines in the figure.

**Figure 7:**
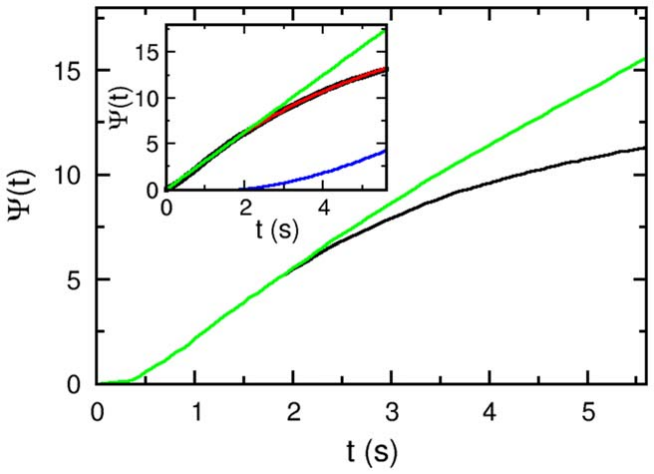
Normalized cumulative distribution functions (CDFs). The measured CDF (black) can be mimicked by the function 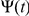 (red), which is given in short times (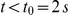) by 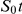 and in long times (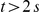) by 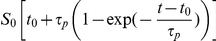, where 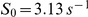 is the initial switching rate, and 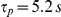. The experimental data after the photodamage correction 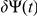, which is represented by the blue line, yields the green line. In the main figure, the same correction function 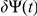 is applied to the measurement (black) when 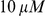 of serine is present. The resulting curve is presented in green.

As a control, we filled the micropipette with the motility buffer (TMN) without serine. The total number of bacteria in this data set was 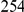, resulting in 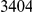 switching events. These events were used to construct the cumulative distribution function, which after normalizing by the cell number is designated as 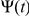. As shown in the inset of Fig. 7, for a short time 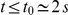, 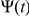 increases linearly with time 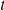, but for 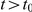, 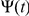 starts to level off in long times. The slope of the initial increase yields the steady-state switching rate 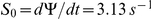, which is consistent with the observation of the free-swimming bacteria (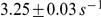) in the steady state (see Materials and Methods). The leveling off of 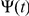 indicates that the bacteria switch less frequently in long times. This is likely due to photodamage, even though most of the cells released after the measurement did not lose their ability to swim. Taking into account this effect, we found that 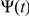 can be adequately described by the following functional form,
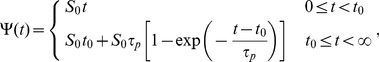
(8)where the characteristic decay time 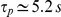. In the inset of Fig. 7, the measured 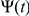 (black curve) is plotted alongside with Eq. 8 (red curve). In the same inset, we also plotted the ideal case (green line), when the bacterial switching rate remains constant at all times. This demands a correction (blue curve) of the form,
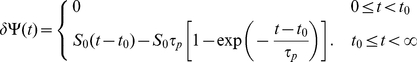
(9)This correction factor 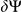 is applied to all of our subsequent measurements with different serine concentrations. An example with 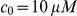 of serine is displayed in Fig. 7, where the measured (black curve) and the corrected 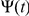 (green curve) are displayed. By definition, the time-dependent switching rate is given by 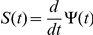. To reduce noises, the data was first binned over the time interval of 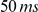 and then a finite difference 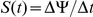 was taken.

**Figure 8:**
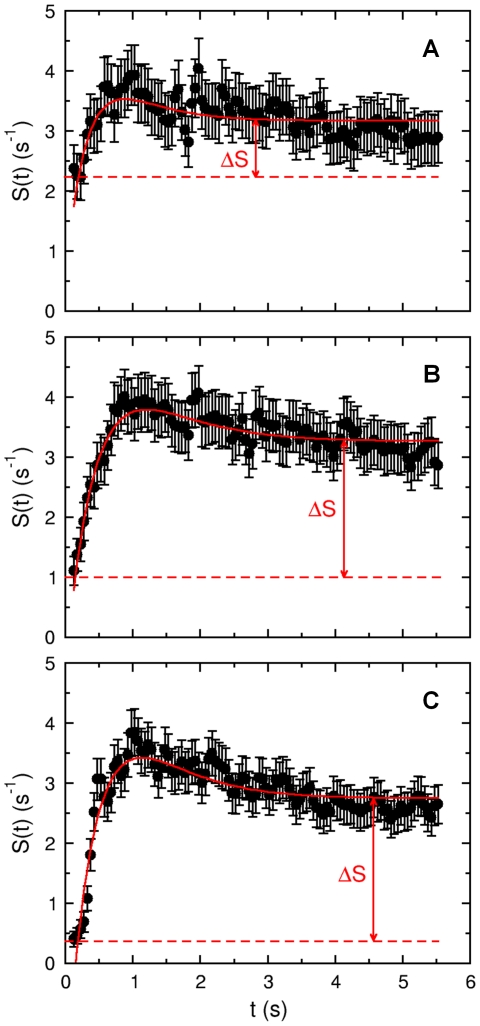
The time-dependent switching rate of *V. alginolyticus*. The measured switching rates for 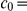
1, 5 and 10 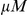 of serine are plotted as dots in (A to C), respectively. The stimulation occurs at 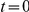. In all the cases, an initial suppression in 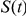 was followed by an overshoot beyond the steady-state switching rate 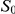. It is only in long times that 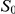 is recovered. The red curves in each plot are the fits using Eq. 15. The fitting procedure yields the following parameters: 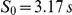, 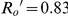, and 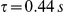 for (A); 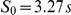, 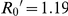, and 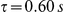 for (B); and 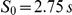, 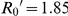, and 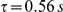 for (C). Here, we treated 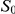 as an adjustable parameter; as can be seen, its value does not change much from run to run.

The time-dependent responses to different levels of chemical stimulations are displayed in Figs. 8(A to C), where the micropipette was filled with 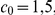 and 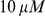 of serine. The number of bacteria in each set was 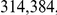 and 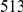 with the corresponding number of switching events being 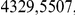 and 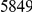, respectively. We noticed that as 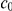 increases, the initial switching rate can be significantly reduced, and in the case of 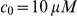, 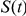 is only 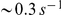 in short times or about a factor of ten less than the steady-state value 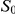. We also noticed that 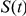 recovers rapidly over time, and the process is biphasic, i.e., 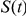 overshoots beyond 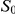 and then relaxes towards 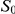 over a long time. Qualitatively, therefore, *V. alginolyticus*' chemotactic response is surprisingly similar to *E. coli*, consisting of a short initial excitation followed by a long adaptive process.

#### D2. Theoretical modeling

The biphasic response was first discovered in *E. coli* [12], and we are surprised to see that *V. alginolyticus* has a similar response. Considerable progress has been made over the past several years in terms of a quantitative understanding of this fascinating behavior in *E. coli* [13], [14], [15], [16], [17], [18], [19]. The progress was made because of extensive knowledge of biochemistry of several *che* gene products and their interactions with chemoreceptors and the motor complex. Although much less is known about *V. alginolyticus'* chemotaxis regulation [20], [21], the similarity in the response seen in our experiment suggests that the regulation mechanism in *V. alginolyticus* may be similar. One of the successful models in explaining the biphasic response is the Monod-Wyman-Changeux (MWC) model proposed by Tu et al. [19]. This mean-field model integrates out fast kinetics of binding and unbinding of chemoeffectors to receptors, and leaves comparatively slow processes of dephosphorylation and methylation as independent variables. The model has been successfully applied to explain the response data acquired in *E. coli* using a variety of stimulation protocols [9], [12]. In the following we will focus on the impulsive stimulation when the serine concentration is low so that the bacterial response may be considered linear.

We assume that the switching rate is determined by the phosphorylated form of response regulator CheY-P whose concentration 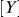 varies with time 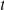 according to,
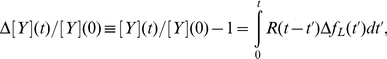
(10)where 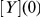 is the CheY-P concentration at the steady state, 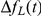 is the change in the free energy (in terms of thermal energy 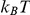) when the ligand concentration varies from its pre-stimulation level 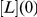 to 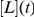, and 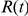 is the linear response (or Green's) function. This assumption is consistent with Kojima et al.'s observation that phosphorylation of CheY is necessary for motor reversals similar to *E. coli* cells [20]. For convenience, we will use *E. coli*'s response function to mimic that of *V. alginolyticus* [19],
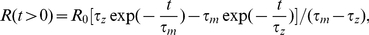
(11)where 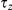 and 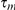 are respectively the dephosphorylation and methylation times, and 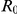 is the amplitude of the response. 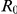 is a measure of the sensitivity of the chemotactic network and is given by 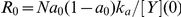, where 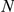 is the number of ligand-binding subunits in the MWC clusters, 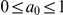 is the average steady-state kinase activity, and 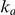 is the phospho-transfer rate, which depends on the total number of MWC complexes in a cell.

For the given dissociation constants of the inactive and the active form of receptors, 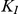 and 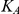, the ligand binding free-energy 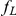 is given by 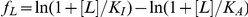. These two dissociation constants also specify a range, 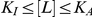, over which the cells are most sensitive to variations in the ligand concentration, and Weber-Fechner law holds approximately [19]. Assuming 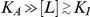 in our experiment, we approximate 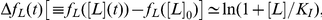 where 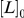 is the background ligand concentration which is assumed to be zero in our experiment. For convenience of presentation below, we will use 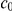 instead of 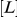 to denote the serine concentration.

The above model enables one to establish the connection between the microscopic chemical-reaction (ligand-receptor binding) kinetics and macroscopic bacterial response. Specifically, we are interested in the switching rate 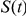 after a brief stimulation by serine. For a weak stimulation, it is reasonable to assume that 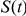 depends linearly on CheY-P concentration such that
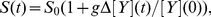
(12)i.e., an increase in CheY-P will increase the switching rate beyond the steady-state value 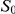. In the above, 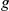 is the gain factor of the motor complex, which is related to the Hill coefficient 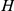 by 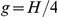. In *E. coli* for instance, 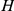 varies from 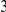 to 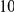 depending on whether the measurements were carried out in an ensemble or in single cells [22], [23]. However, since nothing is known about how the motor complex responses to a change in CheY-P in *V. alginolyticus*, we will set 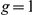. We note that 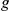 only affects the amplitude of the response function but not its overall functional form. The effect of 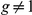 can be readily taken into account once its value becomes available. Substituting 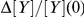 from Eq. 10, we find
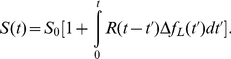
(13)This mathematical result will be compared to our measurements. We noticed that within the linear-response approximation, Eq. (11) implies the adaption is precise, i.e. for a step stimulation 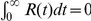, and for a sufficiently long waiting time, 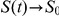. This behavior appears to be consistent with our observations in Fig. 8. As the stimulation in our experiment is brief with an exposure time 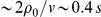, 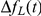 will be approximated by a 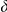 function: 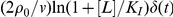. This leads to,
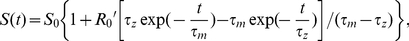
(14)where 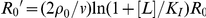. This equation contains three adjustable parameters, 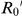, 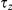, and 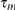, if 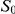 is assumed to be known. While analyzing the data, we found that the best result could be attained when 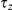 and 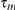 were very close for all of our measurements. In the limit 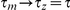, the above equation can be cast in the form,
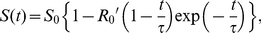
(15)and effectively only two parameters, 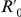 and 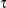, are necessary. As shown in Figs. 8(A to C), all of our data can be fit reasonably well by the above equation, which is indicated by the red lines in the figure. For 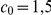, and 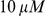, the following results are obtained: 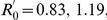 and 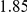, and 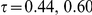, and 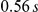. However, considering the large noise in the data, these fittings are not perfect particularly in long times.

The biphasic response is a hallmark of an adaptive behavior. What is unusual in our finding is that the adaptive time is so short that it is indistinguishable from the excitation time. Several lines of evidence showed that *V. alginolyticus* can adapt to serine after a step change 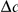 in the serine concentration [24]. The adaptation time becomes longer as 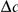 increases. Although a more detailed and quantitative study is needed, this adaptive behavior appears to be similar to *E. coli*. Thus, the short adaptation time seen in our experiment may correspond to either 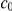 is low or the stimulation is short. In any event, it suggests that *V. alginolyticus* are able to adapt to a wide range of chemical stimulations, which may be significant for bacteria to thrive in the presence of ephemeral micro-scale nutrient sources.

### E. Sensitivity of *V. alginolyticus* to serine

**Figure 9:**
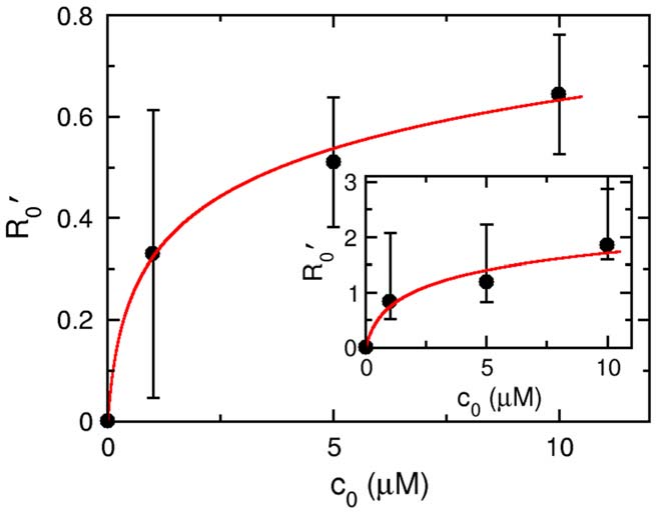
The response amplitude 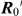 **vs. serine concentration** 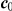. The solid circles are experimental data and the line is the theoretical expression 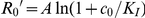, where 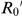 are extracted from Figs. 8(A to C). The fitting procedure yields 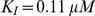 and 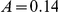. The curve in the inset is generated from the best fit values 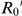 in Fig. 8, and the data can be explained by 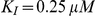 and 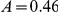, which is plotted as a solid line.

A quantity of significance to bacterial chemotaxis is the dissociation constant 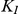. For *E. coli* cells, previous measurements showed 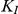 for serine is 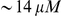 [25]. Our experiments also allow us to estimate 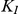 for *V. alginolyticus*. Using the definition 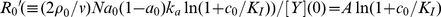, we plotted 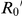 vs. 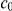 in the inset of Fig. 9, where 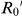 was obtained from the curve-fitting procedure (see Fig. 8). The error bars were calculated based on uncertainties in the measured switching rate 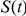. The solid line in the inset of Fig. 9 is the theoretical prediction, where 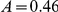 and 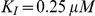 were used. Alternatively one can find 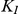 via the relation 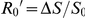 derivable from Eq. 15, where 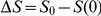 can be easily found for each 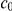 by visual inspection without the fitting procedure. 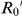 determined in this manner (see Fig. 8) is plotted in Fig. 9, yielding 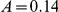 and 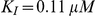. As can be seen, there is a considerable uncertainty in the determination of 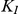 due to the noise in 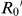. However, it is evident that 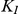 in *V. alginolyticus* is considerably smaller than *E. coli*. Since at 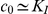 there is only 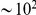 serine molecules in a cell volume and the integration time 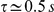 is rather short, it raises the interesting possibility that the threshold of chemosensing in this marine strain may be limited by thermal fluctuations [26], [27], [28].

Finally, using the average value of 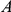, 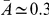, we can estimate the amplitude of the response function 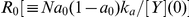 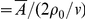, which turns out to be 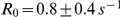. If the gain factor 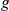 is considered in Eq. 12, 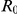 will be reduced by the same factor.

## Discussion

In summary, studies of bacterial chemotaxis have significantly advanced our understanding of how a microorganism interacts with its environment and have general implications for higher level animals that use more sophisticated sensing apparati [29], [30]. Over the last 40 years, methods have been developed to quantitatively investigate this fascinating phenomenon in a variety of bacteria, including *E. coli*, *Bacillus subtilis*, and *Rhodobacter sphaeroides* [31], [32], [33]. The most notable is Adler's modern implementation of the capillary assay that allowed scientists to establish for the first time the existence of specific receptors on bacterial surfaces that play an important role in modulating cell's motility [34]. Berg invented an impressive tracking microscope, which elucidated how *E. coli* cells perform chemotaxis [35]. Silverman et al. developed the rotation assay by tethering a flagellum on a coverslip and observing the rotation of the cell body [6]. This seemingly simple experiment, aside from demonstrating that the flagellum is powered by a rotary motor at its base [6], [36], paved the way for more advanced implementation by conjugating a small bead to the flagellum using antibodies. The rotation assay allowed scientists to study a variety of problems ranging from a torque-speed relation [2], noises in flagellar motor [37], chemotactic responses [9], [12], and molecular interactions between the chemotactic regulatory protein and the motor complex [22], [38]. Herein we added to this impressive arsenal a new approach that allows the cell to be localized while its flagellum and cell-body rotations can be monitored. Similar to the rotation assay, our method is single-cell based and permits the study of behaviors of individual cells as well as the average behavior in a population. A distinctive advantage of our technique is that it does not rely on cell tethering and thus generally applicable to different bacteria. The ease by which the bacterium can be moved by the optical trap also allows one to design “swimming” paths so that complicated memory effects may be studied. The technique when combined with a microfluidic device would allow investigators a great deal of freedom to explore different types of chemical stimulations [39], [40].

Using the optical trapping technique we have investigated *V. alginolyticus*' response to a short pulse of serine. If the regulatory network is linear, the measured response function can be used to interpret bacterial chemotactic behaviors in complicated chemical environments. However, the extent of this linear regime has yet to be established in future experiments. We found that the response function of *V. alginolyticus* is biphasic similar to *E. coli*, suggesting that such a behavior may be evolutionarily conserved. Unlike *E. coli*, however, the putative methylation time 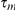 turns out to be so short that nearly matches the dephosphorylation time, 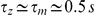. Thus, the chemotactic response of *V. alginolyticus* to a short pulse of attractant essentially consists of only a single time scale. The fast adaptation seen in *V. alginolyticus* is likely due to their habitat where nutrients are short-lived so that unless the microorganisms can recover from the initial excitation quickly, the signal would be lost.

## Materials and Methods

### Cell culture

The bacterial strain *V. alginolyticus* YM4 (Pof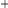, Laf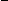) was a kind gift of Michio Homma. The cells were grown overnight in 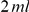 of VC (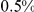 polypeptone, 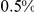 yeast extract, 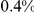 potassium phosphate dibasic, 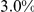 sodium chloride, 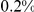 glucose) at 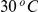 with shaking at 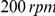. The overnight culture was then diluted 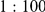 in VPG (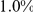 polypeptone, 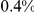 potassium phosphate dibasic, 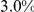 sodium chloride, 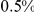 glycerol) and incubated for 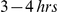 at 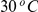 with shaking at 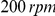. For chemotaxis studies, the cells were washed twice in TMN motility buffer (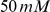 Tris-HCl (pH 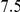), 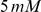 magnesium chloride, 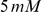 glucose, 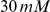 sodium chloride, 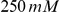 potassium chloride) by gentle centrifugation (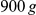, 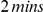) and resuspended in fresh TMN. Based on the swimming speed and the fraction of swimming cells, we found that the optimal incubation time in TMN should be at least 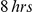 before measurements. For optical trapping, the bacteria were diluted 1∶100 to avoid multiple cells being captured during a measurement.

### Optical trap

The optical trap was formed by focusing an IR laser (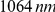, 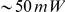 at the laser output) into an open-top chamber with a 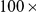 oil immersion objective (see Fig. 2A). Our setup is also equipped with a CCD camera (MTI, CCD72) that enables the bacterial size and the swimming speed 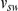 to be measured after the cell being released from the trap. To avoid hydrodynamic interactions with boundaries, the cells were trapped at 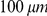 above the bottom surface of the chamber. The trapped bacterium is aligned with the optical axis as depicted in Fig. 2(C to F). The position of the bacterial body in the trap was determined by a silicon position-sensitive detector (PSD) (Pacific Silicon Sensor, DL100-7PCBA) and was digitized at 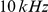 with a 12-bit resolution (National Instruments, AT-MIO-16E-2).

To stimulate a trapped cell, a micropipette was mounted on an x–y stage that held the sample chamber. In this way, the micropipette can move together with the chamber while the optical trap remains fixed in space. The simultaneous movement of the sample chamber and the micropipette relative to the trap is crucial, because in this way the chemoattractant profile remains unperturbed. The x–y movements were controlled by DC actuators (Newport, 850A) whereas the z movement was controlled by a piezo-actuator (Physik Instrumente, P.841.60). Both the x–y and the z actuators are interfaced to a PC via a data acquisition board (National Instruments, AT-MIO-16E-2). The computer controlled x–y and z movements make possible to automate our measurements, which will be discussed in **Measurement procedures**.

When a bacterium swims far from a boundary, its body wobbles around the swimming axis and can be readily seen by optical microscopy. Such a wobbly motion can be a result of a slight asymmetry between the flagellum and the cell body axes or the length of the cell body being not an integer multiple of the half wavelength that the cell body undulates because of flagellum rotation. This wiggly motion manifests itself in the optical trap as well and allows us to simultaneously determine the cell-body and the flagellum rotation angular frequencies, 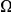 and 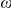, as a function of time 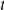 as delineated in Fig. 3 [3]. However, for a highly symmetric cell, the 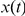 and 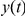 signals in the detector become small, making a motor reversal hard to detect. To make our measurement reliable, the trap beam is slightly tilted as depicted in Fig. 2G. In this case, a cell trapped in the tail-up position with its flagellum rotating CCW is stabilized slightly below the beam waist, causing a small shift in its 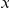-position towards the positive side. Likewise if the flagellum is rotating CW, the cell body will be shifted towards positive 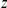 and its 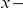
position will be slightly negative. Thus, depending on rotation directions, the cell will be preferentially located in the +x and −x positions in the optical trap, which is seen by the two lobes in Fig. 3A and Fig. 5(A and B). The tilted optical trap significantly improves the detection efficiency of motor reversals.

A drawback of our current setup is that a bacterium can be trapped either in the tail-down (C and E) or tail-up (D and F) configurations as displayed in Fig. 2. For each of these configurations, the cell can swim forward (C and F) or backward (D and E), leading to four possibilities. Even though one can measure 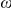 and 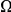, (C) and (D) or (E) and (F) are degenerate, i.e., an experimenter cannot tell if the cell is swimming forward or backward. This degeneracy persists even when the optical trap is tilted. As a result of this deficiency we were only able to measure the bacterial switching rate 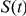 but not the CCW bias.

### Creation of a defined chemoattractant profile

We created a sharp concentration gradient using a micropipette prepared by a microelectrode puller (Narishige, PP-830). The inner diameter of the micropipette is less than 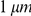 so that bacteria cannot accumulate inside the capillary. The micropipette was filled with serine of concentration 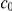 up to a level beyond which a capillary effect vanishes. A small hydrostatic pressure was applied via a plastic tubing by a water column of height 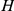 as illustrated in Fig. 2. To calibrate the serine profile, fluorescein dye at 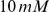 concentration was used, and the fluorescent intensity profile was measured using an electron-multiplying CCD camera (Hamamatsu, C9100-12) and analyzed by SimplePCI (Compix Inc.). We found that for a given 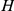, the concentration profile can be established almost instantaneously, in less than 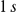, and it is stable over a long period of time, indicating that a quasi-steady state has been reached.

**Tracking of free-swimming cells:** For comparison with the steady-state measurements in the optical trap, we also collected switching statistics of free-swimming cells. The bacterial swimming trajectories were observed under an inverted microscope (Nikon, TE300) with a 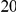
× objective. The cells were confined between two glass coverslips with a spacing 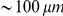. Video images were captured at 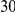 fps by the CCD camera, and the images were analyzed using ImageJ (National Institutes of Health). The mean forward 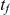 and backward 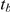 swimming times were measured using an ensemble of 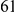 cells, totaling of 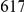 switching events. This yields the mean switching rate 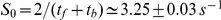, which is consistent with the observation made in the optical trap.

### Measurement procedures

For the time-dependent switching rate measurements, the optical trap was positioned 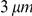 away from the serine-filled micropipette using the computer controlled x–y stage. For an appropriate cell concentration, 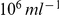, the typical waiting time was about 5 minutes before a single *V. alginolyticus* was captured by the optical trap. Too high a cell density increased the chance that multiple cells would fall into the trap during a measurement; such events were discarded from our data set. Since timing is important in this measurement, the entire procedure was essentially computer controlled. We found that the optical signal detected by the PSD was quiescent when no bacterium was present in the trap. However, when a bacterium was captured, the signal 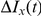 and 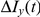 fluctuated wildly, where 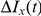 and 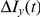 are proportional to the cell-body displacement 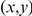 with respect to the trapping center. A typical event is registered in a time series depicted in Fig. 2B, where we noticed that a swimming cell fell into the trap rapidly within 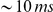, causing a large spike in 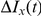. Once the cell became stably trapped, 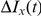 fluctuated with a large amplitude and frequency. The red line in 1B indicates the moment just before the bacterium fell into the trap. We used the rms value of 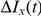 to monitor the status of the optical trap. If the rms value surpassed a pre-determined threshold, the movement of the x–y stage started and this defined 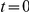 in a measurement. In the subsequent episode, the bacterium was forced to move against the chemical gradient direction (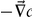) for 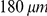 in 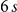 while the rotation of the flagellar motor was continuously monitored by the PSD. The speed of the movement, 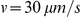, was close to the swimming speed 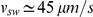 of the bacterium. One can define a chemical exposure time 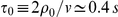, where 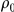 is the characteristic width of the concentration profile. For each serine concentration in the micropipette, at least a few hundreds of bacteria were trapped, resulting in several thousand switching events.
